# Dominant Repression by *Arabidopsis* Transcription Factor MYB44 Causes Oxidative Damage and Hypersensitivity to Abiotic Stress

**DOI:** 10.3390/ijms15022517

**Published:** 2014-02-13

**Authors:** Helene Persak, Andrea Pitzschke

**Affiliations:** Department of Applied Genetics and Cell Biology (DAGZ), University of Natural Resources and Applied Life Sciences (BOKU), Muthgasse 18, Vienna A-1190, Austria; E-Mail: helene.persak@boku.ac.at

**Keywords:** abiotic stress, mitogen-activated protein kinase (MAPK), reactive oxygen species, MYB, repression motif, *Arabidopsis*

## Abstract

In any living species, stress adaptation is closely linked with major changes of the gene expression profile. As a substrate protein of the rapidly stress-induced mitogen-activated protein kinase MPK3, *Arabidopsis* transcription factor MYB44 likely acts at the front line of stress-induced re-programming. We recently characterized MYB44 as phosphorylation-dependent positive regulator of salt stress signaling. Molecular events downstream of MYB44 are largely unknown. Although MYB44 binds to the MBSII element *in vitro*, it has no discernible effect on MBSII-driven reporter gene expression in plant co-transfection assays. This may suggest limited abundance of a synergistic co-regulator. MYB44 carries a putative transcriptional repression (Ethylene responsive element binding factor-associated Amphiphilic Repression, EAR) motif. We employed a dominant repressor strategy to gain insights into MYB44-conferred stress resistance. Overexpression of a *MYB44-REP* fusion markedly compromised salt and drought stress tolerance—the opposite was seen in *MYB44* overexpression lines. *MYB44*-mediated resistance likely results from induction of tolerance-enhancing, rather than from repression of tolerance-diminishing factors. Salt stress-induced accumulation of destructive reactive oxygen species is efficiently prevented in transgenic *MYB44*, but accelerated in *MYB44-REP* lines. Furthermore, heterologous overexpression of *MYB44-REP* caused tissue collapse in *Nicotiana*. A mechanistic model of MAPK-MYB-mediated enhancement in the antioxidative capacity and stress tolerance is proposed. Genetic engineering of MYB44 variants with higher trans-activating capacity may be a means to further raise stress resistance in crops.

## 1. Introduction

### 1.1. Transcription Factors

Transcription, the initial step at which genes are selected for expression, is essential for the regulation of virtually any biological process in living organisms. By binding to their cognate promoter elements, transcription factors (TF) specifically activate or repress expression of respective target genes. TF activity often depends on developmental or exogenous stimuli and/or the presence of co-regulatory proteins. Thus, living organisms can respond to changes in their environment in a highly specific and flexible manner.

### 1.2. Transcription Factors Involved in Plant Stress Responses

Over the last two decades substantial progress has been made in plant stress research. Extensive knowledge exists on TFs that mediate stress adaptation responses in various species. TFs are able to control stress resistance both in homologous and heterologous expression systems are particularly attractive for genetic engineering of stress-tolerant crops [1,2].

Many TF genes show a stress-inducible expression profile, suggesting that various transcriptional regulatory mechanisms function in stress signal transduction pathways [3]. Stress-inducible TFs in plants include members of the DRE-binding protein (DREB) family, the ethylene-responsive element binding factor (ERF) family, the zinc-finger family, the WRKY family, the basic-domain leucine zipper (bZIP) family, the NAC family, the basic helix-loop-helix (bHLH) family, the homeodomain TF family, and the MYB family [3,4]. Ample evidence exists that overexpression of a single TF gene can improve tolerance to diverse, and often multiple, types of stress [5]. For instance, ectopic expression of the ERF gene, *Tobacco stress-induced gene 1 (Tsi1)*, enhanced resistance to pathogen and osmotic stress in tobacco [6]. Overexpression of *AtDREB1A* protected *Arabidopsis* from drought, high salinity and freezing stress [7]. It also improved abiotic stress tolerance in transgenic rice and soybean [8,9]. Several bZIP proteins are involved in signal transduction triggered by ultraviolet light (UV), salt and drought stress. For instance, biotic as well as abiotic stress responses are controlled by bZIP factor VIP1 [10–12]. Similarly, WRKY proteins modify plant resistance by binding to promoters of diverse stress-related genes. *Botrytis cinerea*-induced ethylene biosynthesis relies on direct activation of *ACS2/ACS6* expression by WRKY33 [13]. Recently, *Arabidopsis WRKY22* was shown to function in submergence-conferred immunity [14].

Continuous and aberrant expression of stress-responsive genes leads to substantial changes in the composition of proteins, metabolites and structural components. In the absence of stress, this energy-demanding cellular re-modeling is disadvantageous and frequently correlates with retarded growth. Therefore, TF activity is often controlled in a stress-dependent manner. In many cases, this is achieved via post-translational modifications. For instance, WRKY33 [15], VIP1 [10], ERF6 [16] and C2H2-type zinc finger protein ZAT10 [17] are phosphorylated and regulated by stress-activated kinases.

### 1.3. Transcriptional Activators and Repressors

Transcription factors constitute a major fraction of an organism’s proteome. In *Arabidopsis*, approximately 6% of the protein repertoire are TFs [18], an estimated 30% of which function as transcriptional repressors [18,19]. EAR motifs, which adhere to the consensus LxLxL or DLNxxP, are the most predominant transcriptional repression motifs so far identified in plants [19]. A genome-wide bioinformatics analysis allowed identification of the complete repertoire of the “EAR repressome” in *Arabidopsis* [18]. It comprises 219 (49 experimentally verified, 170 putative) negative regulators of transcription, distributed across 21 TF families. The EAR motif sites are mainly found in the *C*-terminal region [18]. EAR motifs owe their name to the class of TFs in which they had been originally identified (Ethylene responsive element binding factor-associated Amphiphilic Repression (EAR) motif [20]). EAR motifs were shown to function as dominant repressor domains also in other types of TFs, including B3-domain [21,22], MADS-box [23], AUX/IAA [24] and NIMIN proteins [25]. In the MYB TF family, trans-repressing activities could be ascribed to subgroup S4, comprising MYB3, MYB4, MYB7 and MYB32 [26–28], all of which carry an EAR motif. AtMYB4 controls sinapate ester biosynthesis in UV-exposed plants [28], whereas AtMYB32 is required for pollen development [26]. EAR motifs likely mediate repression by physical interaction with other co-repressors [23,29]. According to a current model, EAR repressors regulate gene expression by recruiting chromatin remodeling factors [19]. Phosphorylation and ubiquitination in turn may influence EAR repressor function and turnover [19]. Interestingly, when tethered to an EAR motif, transcriptional activators can be converted into dominant repressors [30]. The DNA-binding specificity in such chimeric proteins is maintained. The EAR fusion repressor technology has therefore been proposed as a unique opportunity to overcome the challenges of gene redundancy [30].

### 1.4. *Arabidopsis* MYB Transcription Factors—Subfamily S22

MYB-type transcription factors form large protein families in eukaryotic species. In plants, diverse developmental processes and physiological responses are mediated by MYB proteins. A distinct subfamily of these TFs is essential for activating phenylpropanoid biosynthesis [31–34]. MYB proteins also act as positive or negative regulators of stress signaling (see below). In addition, MYB transcription factors are promising candidates for engineering of stress-tolerant crops [2].

The plant R2R3 MYB family is particularly large, comprising 126 members in *Arabidopsis* [35]. R2R3 MYB proteins carry a characteristic, highly conserved *N*-terminal DNA-binding domain and a variable *C*-terminal region comprising an activation or repression domain. Based on the conservation of the R2R3 region and of motifs in the *C*-terminal part, *Arabidopsis* R2R3-MYB proteins can be divided into 23 subgroups [35]. Most MYB proteins bind to one or more of two types of *cis*-elements, known as MYB binding sites (MBS), the consensus sequences of which are CNGTT(A/G) (MBSI), C(G/T)T(A/T)GTT(A/G) (MBSII) [36].

MYB subfamily S22 comprises four members, MYB44, MYB70, MYB73 and MYB77. MYB77 functions in auxin signaling and the control of lateral root formation [37]. MYB44 mediates responses to aphid attack [38], high salinity and drought stress [39–41]. *MYB73* expression is induced by volatiles of two growth-inhibiting rhizobacteria [42]. Protein functions of MYB70 and MYB73 are still obscure. As we have shown recently [40], members of the S22 family are phosphorylated *in vitro* by MPK3, a mitogen-activated protein kinase that controls a diversity of abiotic and biotic stress responses [43–46]. In MYB44, the MPK3 phosphorylation site was located to a single residue (Ser145) [40]. Knowledge about MPK3 docking domain(s) in MYB44 is based on *in silico* predictions only. Ser145 and flanking regions seem to be conserved within the S22 family. Mutation at S145 does not affect MYB44 nuclear localization nor its DNA-binding properties [40]. MYB44 binds to MPK3 *in vivo*; and it also forms homodimeric MYB44/MYB44 complexes [40]. These protein-protein interactions are nuclear-located and independent of the phosphorylation state at Ser145. Overexpression of *MYB44* rendered transgenic *Arabidopsis* seedlings more tolerant towards salt stress. On the contrary, reduced tolerance was observed for seedlings overexpressing a MYB44 de-phosphomimetic (Ser145Ala) mutant variant. S145Ala molecules may block critical domains in MPK3 or prevent endogenous MYB44 and/or its closest homolog, MYB77, from binding to cognate DNA elements [40].

Molecular factors and mechanisms that act downstream of *MYB44* in stress signaling are largely elusive. Here, we investigated the mode of MYB44 action as transcriptional regulator and its possible implication in redox balancing under stress conditions. We sought to manipulate the trans-regulatory activity of MYB44. Our motivation was to propose protein modifications that potentially further accelerate MYB44-mediated stress resistance. This was also done in view of possible biotechnological applications, since ectopic expression of *AtMYB44* has been shown to improve salt and drought stress tolerance in soybean [41]. We asked whether the positive effect of *MYB44* in stress-exposed plants was primarily due to transcriptional activation or repression of target genes.

## 2. Results

### 2.1. MYB S22 Family Members Carry a Putative Repressor Domain

**Figure 1.**
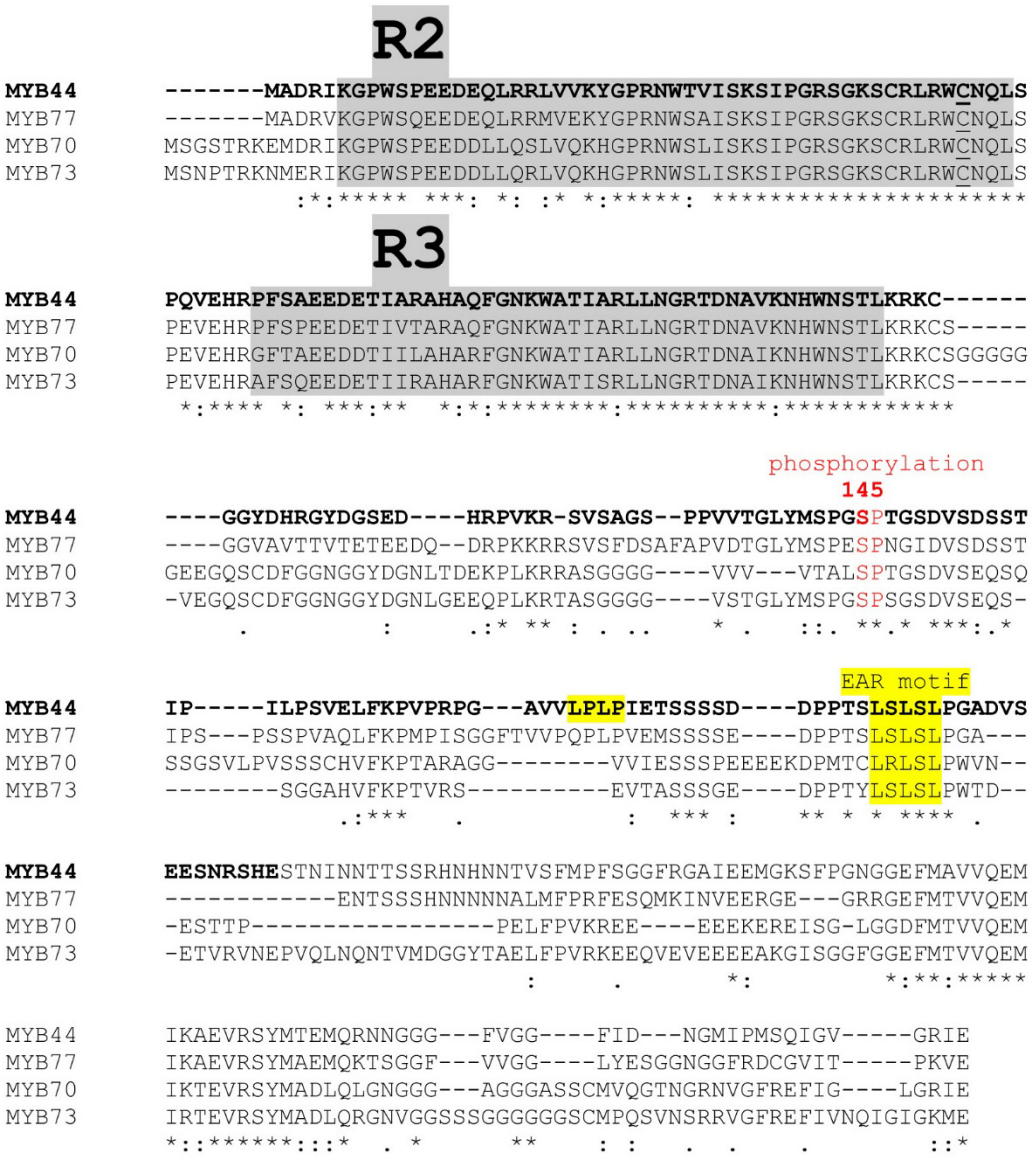
Protein sequence alignment of *Arabidopsis* R2R3 MYB subfamily 22. The R2R3 repeats of the DNA binding domain (grey) and putative transcriptional repressor motif (yellow) are highlighted. The known MPK3 phosphorylation site in MYB44 (Ser145) is indicated. The *N*-terminal (aa1-212) MYB44 region known to bind particularly strongly to DNA (MBSII element) is shown in bold. A conserved cysteine residue potentially involved in redox-dependent MYB activity is underlined. “.” “:” and “*” mark residues with low, moderate and high conservation.

The primary protein sequence alignment of R2R3 MYB subfamily S22 reveals a short peptide stretch that is conserved in all four members. This region adheres to the consensus of EAR motifs (LxLxL) (Figure 1). In addition, MYB44 contains a second peptide (vLPLPi) (Figure 1), which resembles a partial EAR. Accordingly, MYB44, MYB77 and MYB70 are among the 219 candidate proteins potentially constituting the “*Arabidopsis* repressome” [18].

**Figure 2.**
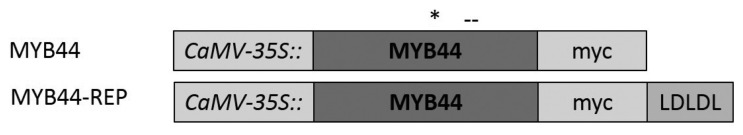
Schematic illustration of DNA constructs used for overexpression *in planta*. Relative positions of MPK3 phosphorylation site (*) and the putative endogenous EAR motif (--) are indicated.

We aimed to investigate a possible role of the MYB S22 proteins as transcriptional repressors, using MYB44, the best-characterized member of this subfamily. In order to exclude/minimize possible interference with MPK3 binding, we chose not to mutate or delete the endogenous putative EAR motif in the MYB44 protein. Instead, an artificial EAR motif was introduced into MYB44 by fusing a LDLDL peptide to the *C*-terminal end of the protein. The resultant chimer (named MYB44-REP) was placed under control of the CaMV35S promoter (Figure 2) and ectopically expressed in stable transgenic *Arabidopsis* plants. As shown previously, transgenic *Arabidopsis* lines carrying a similar construct, CaMV35S::MYB44, are more tolerant towards osmotic stress [40] consistent with an earlier study by Jung *et al.* [39]. The identity and direction (activation or repression) of the corresponding stress-responsive MYB44 target genes are still unclear. Two scenarios can be imagined: (i) If MYB44 acted as transcriptional activator of osmotic stress-tolerance-enhancing genes, a hypersensitive phenotype might be expected for *35S::MYB44-REP* plants; and (ii) If MYB44 actively repressed negative regulators of stress tolerance, *MYB44-REP* overexpression should improve tolerance.

**Figure 3.**
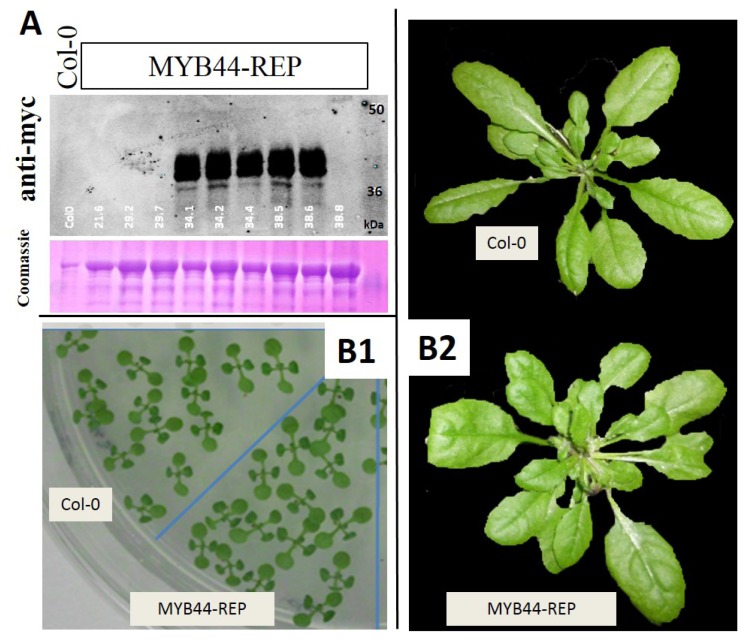
Stable expression of MYB44-REP in transgenic *Arabidopsis* plants. (**A**) Documentation of overexpression. Eight-day-old seedlings of Col-0 and MYB44-REP transgenic lines were separated by SDS-PAGE. Transgene expression was visualized by immunodetection, using rabbit anti-myc and IRDye800CW-coupled donkey-anti-rabbit (NEB, Frankfurt, Germany) as primary and secondary antibodies, respectively. Strong transgene expression can be seen in five lines; and (**B**) MYB44-REP overexpression in *Arabidopsis* does not interfere with normal development; (**B1**): 10-day-old seedlings grown on standard medium; and (**B2**): Aerial views of rosettes of 5-week-old plants of wild-type and MYB44-REP overexpressing plants. Images of one representative line, which was phenotypically indistinguishable from other MYB44-REP lines, are shown.

*MYB44-REP* transgenic lines were generated and propagated into a homozygous state. To allow maximal comparability, transformation and propagation was carried out in parallel with 35S::MYB44 lines. Transgene expression was verified by immunoblot analysis using an antibody directed against the (*C*-terminal) myc epitope tag (Figure 3A).

### 2.2. *MYB44-REP* Overexpression Interferes with Early Stress Responses and Antioxidative Defenses in Salt-Exposed *Arabidopsis* Seedlings

**Figure 4.**
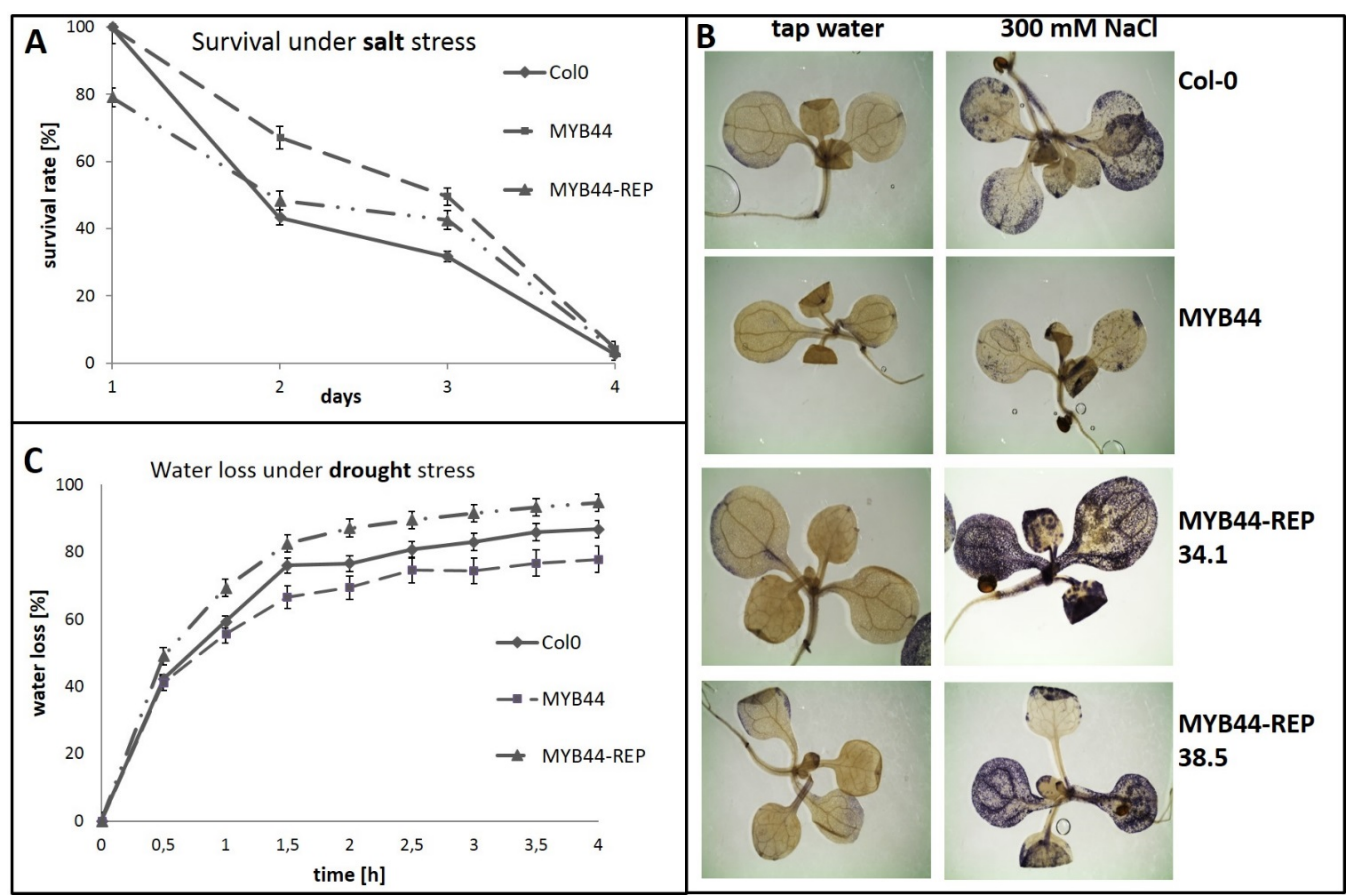
*MYB44-REP* overexpression compromises abiotic stress tolerance. (**A**) Survival under high-salinity conditions. Three-day-old seedlings grown under standard conditions were transferred onto growth medium supplemented with 150 mM NaCl. Seedling survival was monitored over a 4-day period. About 50 seedlings of 4–6 independent lines each were tested. Results of two independent experiments are shown; (**B**) Superoxide accumulation upon osmotic shock treatment. Twelve-day-old seedlings were incubated in tap water with or without 300 mM NaCl for 6 h. Superoxide radicals were visualized by nitro blue tetrazolium (NBT) staining. Five seedlings per treatment per line were tested in parallel; one representative image is shown. The experiment was repeated twice; and (**C**) Drought stress. Roots were detached from 3-week-old seedlings that had grown on standard medium. Water loss was determined at regular intervals over a 4 h period. The experiment was repeated twice.

We studied the physiology of confirmed transgenic lines as well as their tolerance to osmotic stress, using the experimental conditions established in our previous study [40]. Under standard conditions, germination and growth of *MYB44-REP* lines were similar to that of control plants (Col-0) (Figure 3B). Thus, *MYB44-REP* overexpression in *Arabidopsis* has no apparent effects on plant development. In contrast, under high-salinity conditions (three-day-old seedlings, transferred onto 150 mM NaCl), fundamental differences became apparent (Figure 4A). All seedlings of *MYB44* and Col-0 survived the first day on salt-containing medium. Consistent with our earlier report [40], stress symptoms appeared during the subsequent two days; and seedling survival was higher in *MYB44* lines, compared to Col-0. In contrast, 20% of *MYB44-REP* seedlings died already during the first day of salt exposure. Interestingly, the reduced tolerance in *MYB44-REP* lines was confined to day one. Furthermore, the data suggest that ectopically expressed *MYB44* can delay, but not prevent salt-induced seedling death (under the tested conditions). Reciprocally, *MYB44-REP* expression appears to accelerate tissue collapse. Histological staining of seedlings after 6 h of salt treatment further support that MYB44-REP interferes with rapid stress responses (Figure 4B).

Numerous biotic and abiotic stresses provoke the formation of reactive oxygen species [47,48]. Cellular damage caused by ROS production can be prevented to some extent through the action of ROS scavenging enzymes. Superoxide (O_2_^−^) is particularly aggressive. To determine whether the different salt stress phenotypes exhibited by *MYB44* and *MYB-REP* lines (Figure 4A) were attributable to altered superoxide accumulation, seedlings were exposed to 300 mM NaCl for 6 h and subsequently stained with nitro blue tetrazolium (NBT). NBT is reduced by superoxide to blue, water-insoluble formazan. Untreated seedlings of all lines contained some basal O_2_^−^ levels, which did not markedly differ (Figure 4B). All lines responded to the high-salt treatment, visible by increased formazan precipitation. Notably, *MYB44* seedlings accumulated less O_2_^−^ than Col-0. The opposite was observed for *MYB44-REP* seedlings. These findings imply that (i) *MYB44* overexpression improves salt tolerance by inhibiting ROS formation and/or by activating ROS scavenging mechanisms; (ii) Rapid death in salt-exposed *MYB44-REP* plants is, at least partially, attributable to aberrant superoxide accumulation; (iii) Stress-related events caused by *MYB44* and *MYB44-REP* are opposite; and (iv) *MYB44* appears to be particularly important during early stages of salt stress exposure.

### 2.3. *MYB44-REP* Overexpression Reduces Tolerance to Drought Stress

We further studied *MYB44-REP* overexpression effects on physiological responses associated with *MYB44* function. *MYB44* overexpressing plants are reportedly more tolerant towards drought [41]. Consequently, water loss rates were compared in *MYB44*, *MYB44-REP* and wild type plants under dehydration conditions (Figure 4C). *MYB44* plants exhibited stronger water retention ability, compared to control plants. This is consistent with a recent report [41]. Contrarily, water loss was augmented in *MYB44-REP* lines. Taken together, the results from salt (Figure 4A,B) and drought stress experiments (Figure 4C) point to a role of MYB44 as activator of genes required for abiotic stress tolerance. Active repression of these target genes is destructive to the plant (Figure 7).

### 2.4. MYB44 and MYB44-REP Transactivation Studies *in Vivo*

**Figure 5.**
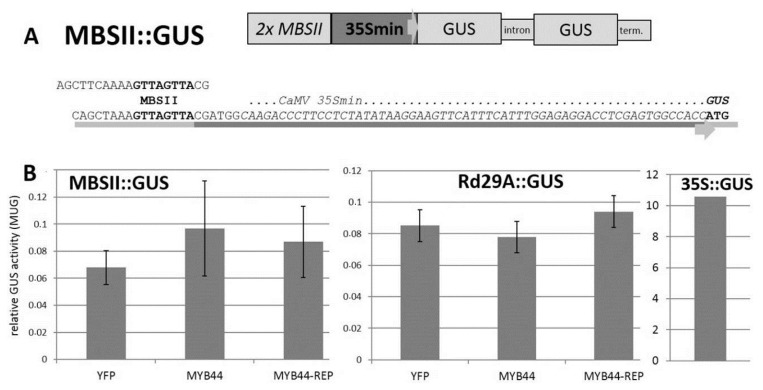
Promoter construct and co-transfection assays. (**A**) Partial sequence of the MBSII-35Smin-GUS construct. The tandem MBSII element is highlighted; the minimal CaMV35S promoter region is underlined. The GUS start codon is shown in bold. For comparison, the DNA fragment efficiently bound by MYB44 [40] is shown in the top line; and (**B**) Promoter/effector relations were studied in *Arabidopsis* protoplasts. *Arabidopsis* mesophyll protoplasts were co-transfected with the reporter constructs MBSII::GUS or Rd29A::GUS, plus one of the following effector constructs: YFP (control), MYB44 or MYB44-REP. β-glucuronidase activity was assessed in protein extracts by quantitative MUG assay. Conversion of the substrate was measured 4 h after incubation at 37 °C. Six aliquots of protoplasts were transformed for each promoter/effector combination. Protoplast transformation of 35S::GUS for constitutive expression of the reporter gene served as positive control. Note that GUS expression from the 35S promoter is approximately 100 times higher than in MBSII or Rd29A. The experiment was repeated twice with similar results.

Next, we aimed to explore the functions of MYB44 and MYB44-REP as transcriptional regulators. Although a microarray study had revealed a number of differentially expressed genes in *MYB44*-overexpressing or -deficient lines [39], it is still unclear which of these are direct and which are secondary targets. MYB44 is known to preferentially bind the MBSII element [49]. *In vitro*, this binding is highly efficient and seemingly unaffected by S145 phosphorylation [40]. A synthetic construct consisting of a tandem MBSII element, the CaMV35S minimal promoter and the glucuronidase (GUS) reporter gene was generated (Figure 5A).

This reporter construct in combination with one of the following effector constructs: control (*YFP*), *MYB44-myc* or *MYB44-myc-REP* was introduced into *Arabidopsis* mesophyll protoplasts or into *Nicotiana benthamiana* leaves via PEG- or *Agrobacterium*-mediated transformation, respectively. As shown previously, in both expression systems a MYB44-YFP fusion protein locates to the nucleus [40]. This is most likely attributable to the nuclear localization signal located at the *N*-terminus. It is therefore reasonable to assume that myc or myc-EAR epitope tags (which are shorter than YFP) do not affect the native localization of the MYB44 protein.

Five days after agrobacterial infiltration, GUS activity was assessed in *N. benthamiana* leaves by histological staining with X-Gluc. In at least six independent repeats of co-infiltration/GUS assay experiments very faint or no blue coloration was detected, and there was no discernible difference in GUS activity between tissue co-infiltrated with *MBSII*::GUS plus *YFP*, *MYB44* or *MYB44-REP* (not shown). UV microscopy of the control sample (YFP) revealed strong fluorescence throughout the infiltrated area, documenting high transformation efficiency in *N. benthamiana* (Figure S1). Also, strong GUS activity was observed in positive control samples (constitutive expression construct, *CaMV35S*::GUS). *N. benthamiana* protein extracts were further analyzed by immunoblotting. Specific immune-reactive bands were found in samples of [MBSII::GUS + *MYB44*] and [MBSII::GUS + *MYB44-REP*], but not in the control [MBSII::GUS + *YFP*] (Figure S1). Therefore, the lack of visible effects of MYB44 or MYB44-REP on MBSII promoter activity unlikely results from impaired expression or instability of the putative effector proteins.

Data obtained from co-transfection studies using an alternate transient expression system corroborate these findings. *Arabidopsis* mesophyll protoplasts were co-transfected with the transgene combinations used in the above-described leaf infiltration experiments. Reproducibly, (independent protoplast isolations, replicate transfections, enzymatic assays), GUS activity in protein extracts from all samples was low. Similarly to *N. benthamiana* (see above), reporter gene expression did not notably differ between samples carrying any of the effector constructs *MYB44*, *MYB44-REP*, or *YFP* (Figure 5B). Parallel protoplast transformations performed with a *CaMV35S*::GUS construct for constitutive expression of β-glucuronidase documented functionality of the assay.

Based on the established involvement of MYB44 in osmotic and drought stress signaling, we investigated the *cis*-regulatory effect of this protein on a (putative direct target) stress-related gene. Selection was based on two main criteria: (i) presence of MBSII motif(s) in the promoter and (ii) well-documented response to abiotic stress. *Rd29A* is a marker gene for abiotic stress. In proximity to the 5′UTR the *Rd29A* promoter contains a “GTTAGTTA” signature which matches a tandem MBSII motif. MYB44 reportedly binds strongly and specifically to this signature *in vitro* [40]. We therefore generated a *Rd29A*::GUS reporter construct for *Arabidopsis* mesophyll protoplast co-transfection studies. The quantitative assay revealed some GUS activity in protein extracts of all samples. The two effectors, MYB44 and MYB44-REP, did not differ in their trans-activating capacity and were similar to the control effector (YFP). In summary, ectopically expressed *MYB44* or *MYB44-REP* had no discernible trans-activating or -repressing effect on a synthetic MBSII promoter or the *Rd29A* promoter.

### 2.5. Ectopic Expression of MYB44-REP Causes Tissue Collapse in *N. benthamiana*

**Figure 6.**
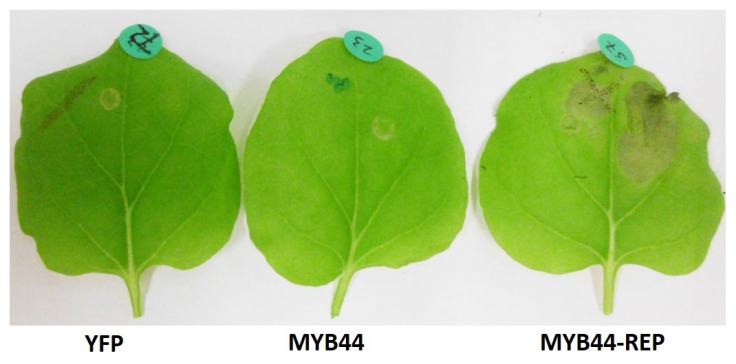
Overexpression of *MYB44-REP* causes necrotic lesions. *N. benthamiana* leaves were infiltrated with Agrobacteria carrying a construct for overexpression of *YFP* (control), *MYB44* or *MYB44-REP*. The photo was taken eight days post-infiltration. All leaves shown are derived from the same plant. Consistent results were obtained from infiltration experiments with individual plants (1 plant/construct).

In the course of *N. benthamiana* co-infiltration studies, a striking impact of ectopic *MYB44-REP* expression on leaf tissue vitality was observed: While leaves expressing *YFP* or the *MYB44* transgene displayed no major symptoms caused by bacterial infiltration, *MYB44-REP*-infiltrated leaves developed severe necrosis. Lesions were visible 3 days after infiltration and became more pronounced later on (Figure 6). Single infiltration experiments—with *YFP*, *MYB44* or *MYB44-REP* only—substantiated these findings and showed *MYB44-REP*-related symptoms to be unrelated to the presence of the MBSII reporter construct. Furthermore, the symptoms were consistently observed in *N. benthamiana* leaves infiltrated at the 14-day seedling or 6-week adult plant stage, indicating that *MYB44-REP*-triggered necrosis was an age-independent phenomenon. Transfection with the de-phosphomimetic *MYB44* variants *S145A* or *S145D* [40], respectively, did not provoke necrosis (not shown). Furthermore, *MYB44-REP*-related necrosis was not prevented or accelerated upon co-infiltration of *MKK4* and/or *MPK3*. Consequently, the symptoms are most probably unrelated to the protein’s Ser145 phosphorylation state.

## 3. Discussion

### 3.1. MYB44-REP-Induced Tissue Collapse in *N. benthamiana*

Transcription factors engineered to encode an EAR motif are able to repress the expression of the target orthologous genes across multiple plant species [50]. A likely explanation for *MYB44-REP*-triggered tissue collapse therefore is an inappropriate repression of genes that are required for cellular homeostasis. Whether this applies to homeostasis in general or to homeostasis under biotic stress conditions remains elusive. Inherent to the experimental system (agroinfiltration), effects of *MYB44-REP* expression cannot be assessed in a pathogen-free context. At this point it is interesting to note that stable overexpression of *AtMYB4* or its ortholog from *Antirrhinum majus*, *AmMYB308*, in *N. tabacum* leads to slower growth and the formation of premature white lesions [28,51]. Both proteins contain an EAR motif and actively repress genes involved in the biosynthesis of sinaptate esters, UV-protecting compounds [28]. The authors also noted a dose-dependent change in the target specificity of AtMYB4 in transgenic *Arabidopsis* plants [28]. In our study, transgene-derived protein levels of MYB44 and MYB44-REP were similar, both in stably transformed *Arabidopsis* lines and infiltrated *N. benthamiana* leaves. The contrary effects caused by *MYB44* and *MYB44-REP* overexpression unlikely result from altered target gene selectivity but from opposite “orientation” of target gene regulation (trans-activation or repression).

### 3.2. Lack of MBSII-Related Transactivation/Repression in Co-Transfection Assays

Protein–protein interactions can significantly impact the regulatory activity of MYB transcription factors [2,52]. MYB proteins with two or more MYB repeats bind DNA *in vitro* as monomers. This is accomplished through cooperative interaction between the tandemly-arranged MYB repeats, which function like “covalently linked” dimers when contacting DNA [53]. Others [49] and we [40] had documented efficient binding of MYB44 to MBSII *in vitro*. However, MYB44 or MYB44-REP had no discernible effect on MBSII-driven reporter gene expression in plant co-transfection assays. This is unlike the situation in stress-exposed transgenic plants, where MYB44 or MYB44-REP gave rise to clear effects (Figure 4). Exposure of *N. benthamiana* co-transfected leaves to salt or drought did not stimulate or repress reporter gene expression (data not shown). Co-regulatory protein(s) might be required for MYB44 to be fully active as transcription factor. Such co-regulator might be limited/absent in the expression systems used, *i.e.*, *Arabidopsis* mesophyll protoplasts and *N. benthamiana* leaves. In the former system, lack of a cell-cell context could be a further limiting factor. In infiltrated *N. benthamiana* leaves, agrobacterial factors might directly or indirectly (by changing host protein properties) prevent binding of MYB44 to the synthetic MBSII motif. Impeded accessibility of a (putative) redox-regulatory factor to the MYB44 protein offers an alternative explanation, particularly since a cysteine residue, shown to be crucial to the conformation and DNA binding activity of the v-myb oncogene [54], is conserved in MYB44 (S43 of R2 repeat, Figure 1).

Transcriptional regulatory activity of some R2R3-MYB factors *in vivo* requires protein–protein interactions. For instance, members of R2R3 subgroup S15 [35] and basic helix–loop–helix (bHLH) proteins [55] cooperatively control gene expression. Associations with bHLH proteins represent the major type of MYB/TF combinations identified so far. Importantly, bHLH interaction is crucial for stable binding of MYB proteins to DNA, as exemplified by TT2/TTG1 a TF pair synergistically regulating tannin biosynthesis [56]. The bHLH interaction signature motif, defined as ((D/E)Lx_2_(R/K)x_3_Lx_6_Lx_3_R) in the R3 repeat occurs in 20 MYB proteins [57–59]. Since *MYB44* or other members of the S22 subfamily do not contain such motif, co-operation with bHLHs similar to those reported seems unlikely. Further modes of synergism between MYB factors and partner TFs in *Arabidopsis* include *MYB30/BES1* in brassinosteroid signaling [60]; *MYB18/FHY1* and *FHL* in phytochrome A signaling [61] and *MYB77/ARF7* in auxin signaling and lateral root development [37].

In conclusion, limited availability of a co-regulator or modifying factor seems the most likely explanation for the discrepancy between MYB44 behavior towards MBSII *in vitro* (strong binding; electrophoretic mobility shift assay [40] and *in vivo* (co-transfection assays, this study). Future research shall concentrate on identifying proteins that engage in a combinatorial transcriptional control with MYB44.

### 3.3. Narrowing down the MYB44 Targetome

Using a microarray approach, Jung *et al.*, 2009 [39] could disclose a large number of genes showing elevated or reduced transcript abundance in MYB44 overexpressing or deficient plants; under standard or high-salinity conditions. This data list likely contains both direct and secondary MYB44 target genes. As suggested by the stress tolerance phenotypes of *MYB44 vs. MYB44-REP* lines (Figure 4), MYB44 more likely acts as inducer of positive stress regulators rather than as repressor of negative stress factors. Therefore, as additional output from the studies of [39,40,49] and the data represented here, the number of putative direct MYB44 target genes may be narrowed down to those that (i) Are rapidly induced under osmotic stress conditions; (ii) Contain MBSII element(s) in their promoters; (iii) Have elevated transcript levels in *MYB44*oe plants and/or (iv) Reduced transcript levels in *myb44* mutants.

The latter criterion is somewhat problematic due to potential redundancy of *MYB44* with *MYB77*. However, the dominant repressor strategy (MYB44-REP) should facilitate verification of suspect target genes in co-transfection studies.

### 3.4. Effects of MYB44 and MYB44-REP on Osmotic Stress Tolerance in *Arabidopsis*

Various methods exist to study osmotic stress responses in plants. Each has its advantages and disadvantages. We mimicked drought and high-salinity conditions by exposing seedlings to desiccation stress or to NaCl-containing medium, respectively. Seedlings are far more homogenous than adult plants, and many individuals/transgenic lines/sublines can be analyzed side-by-side. For both *MYB44* and *MYB44-REP*, six transgenic lines arising from independent T-DNA integration events were studied in parallel. For histologic stainings of ROS accumulation, seedlings underwent a rapid and harsh treatment (6 h, 300 mM NaCl). Of course, the stress exposures used are rather drastic treatments, which certainly cannot fully reflect osmotic stress encountered by plants in their natural habitats. However, these are generally accepted standard techniques which facilitate data comparison between research reports.

Overall, salt and drought stress tolerance was enhanced in transgenic *MYB44* but reduced in *MYB44-REP* lines. The MYB44-REP fusion protein carries a non-mutagenized S145 residue and therefore likely is accepted as substrate for MPK3 phosphorylation. It cannot be excluded that introduction of the additional EAR peptide affected the efficiency of MPK3 binding and/or phosphorylation. However, the peptide is comparatively short (6 residues), located at the *C*-terminus and distant from the S145 phosphorylation site (Figure 2).

Interestingly, *MYB44-REP* seedling survival on salt was lower only at the first day (Figure 4A). *MYB44*-induced stress gene expression therefore seems particularly important in the early stress response. Accordingly, histological studies revealed substantial differences between Col-0, *MYB44* and *MYB-REP* lines within 6 h of salt stress exposure. Superoxide accumulation was repressed in *MYB44*, but enhanced in *MYB44-REP* lines. In summary, from the above-described data and considerations, we come to the following conclusions:

In the absence of stress, phenotypes and superoxide levels in *MYB44* and *MYB44-REP Arabidopsis* transgenics are similar to wild type. TF activity is apparently stress-dependent and most likely regulated by MAPK-mediated phosphorylation. A similar scenario has recently been described for *Arabidopsis* ERF6. H_2_O_2_-activated MPK6 phosphorylates ERF6. The phosphorylated TF subsequently binds to ROS-responsive elements to regulate oxidative gene expression [62].

Under salt stress, (i) *MYB44* functions as transcriptional activator rather than as a repressor. It induces positive stress-regulatory genes; (ii) *MYB44* acts in the early stress response. At later stages, other tolerance-improving regulators may become sufficiently active. These would mask *MYB44*-related positive effects and counteract the destructive effects caused by *MYB44-REP*, respectively; (iii) NBT staining data suggest MYB44 to prevent stress-triggered tissue collapse by rapid removal of destructive superoxide; (iv) This enhanced antioxidative capacity likely results from direct or indirect transcriptional induction of ROS scavenging enzymes; and (v) Salt-responsive MYB44-regulated genes are still elusive; *Rd29A* is an unlikely direct target or requires presence of a co-regulator.

Along with numerous other TFs that have been implicated in salt stress signaling [6,7,12], *MYB44* participates in fine-tuning transcriptome adaptation responses.

Upon heterologous overexpression (*N. benthamiana* agroinfiltration), *MYB44-REP*, but not *MYB44* negatively affects tissue integrity. Therefore the introduced TF does recognize non-*Arabidops*is promoters; and the “direction” of regulation (*MYB44* as activator; *MYB44-REP* as repressor) appears to be maintained across species.

**Figure 7.**
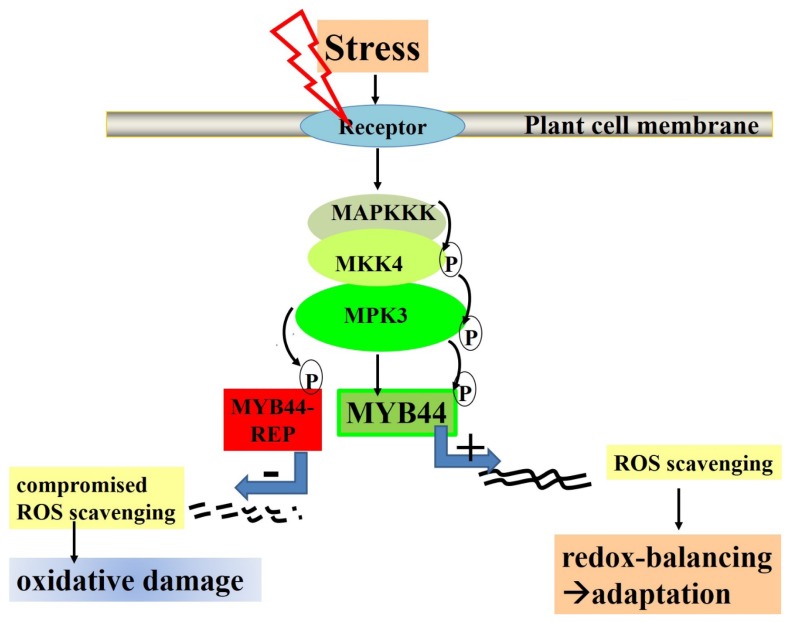
Proposed model of MYB44 function in stressed plants. Upon stress perception, a mitogen-activated protein kinase cascade is activated. MAPKK4 phosphorylates and thereby activates MPK3. MPK3 phosphorylates MYB44, thereby stimulating MYB44 function as transcriptional inducer [40]. **Right**: MYB44-induced target genes, directly or indirectly, enhance the antioxidant capacity, e.g., through activating reactive oxygen species (ROS)-scavenging enzymes. This ultimately leads to a balanced redox state and prevention of cell death; and **Left**: in contrast, dominant repression of MYB44 target genes adversely affects redox-balancing, leading to massive accumulation of destructive ROS.

A model proposing *MYB44* function in stressed plants is outlined in Figure 7. Future studies will focus on whether *MYB44* (and S22 members) are “bivalent” transcriptional regulator(s) that—depending on the type of stress—act as activator or repressor.

## 4. Experimental Section

### 4.1. Plant Material, Osmotic Stress and Drought Tolerance Test

*Arabidopsis thaliana* ecotype Columbia (Col-0) plants were routinuously grown at 20 °C, 60% humidity under long-day conditions (16/8 h light/dark). To break dormancy, seeds were incubated at 4 °C for 2 days. At least 2–3 individual T3 transgenic lines were used for the stress assays. Experiments were independently repeated at least three times. Seeds were surface-sterilized with NaOCl/90% ethanol for 5 min and washed three times with 96% ethanol. Surface-sterilized seeds were incubated on half-strength MS medium/0.25% sucrose/1% plant agar. After stratification for 2 days at 4 °C, plates were transferred to an incubator (8/16 h dark/night regime, 25 °C).

For osmotic stress tolerance tests, seeds were plated on half-strength MS medium (see above). After 3 days, seedlings were transferred to plates containing half-strength MS supplemented with 150 mM NaCl and grown for further 4 days. About 50 seeds were used for each treatment.

### 4.2. Water Loss Determination

Three-week-old seedlings grown under standard conditions were detached from their roots and immediately weighted (fresh weight). The plants were kept at room temperature (on a petri dish) and weighted at the times designated. Finally, plants were completely dried for at least 12 h at 70 °C (dry weight). Water loss was calculated according to the formula of [63].

(1) Water content (WC) [%]=(desiccated weight-dry weight)/(fresh weight-dry weight)×100

### 4.3. Detection of Superoxide

Twelve-day-old seedlings were incubated in tap water with or without 300 mM NaCl for 6 h. Superoxide radicals were visualized by NBT staining (0.1 mM NBT in 20 mM HEPES, pH 7). Stained plants were bleached in a solution containing acetic acid:glycerol:ethanol (1:1:3, *v*/*v*/*v*) at 70 °C. Photographs were taken using a camera connected to a light microscope.

### 4.4. Constructs

All constructs for plant transformation are based on the pGreen/pSoup binary vector system [64]. Plasmids 35S::YFP and 35S::MYB44 have been described previously [40]. To generate 35S::MYB44-REP, a DNA fragment comprising a myc tag and the repressor motif (see Figure 2) was added in-frame to the 3′ end (NotI site) of the MYB44 coding region in 35S::MYB44.

35S::GUSint has been described previously [10]. To construct the MBSII element-reporter plasmid, the VIP1 response element in the previously described VRE1-35Smin::GUSint construct was replaced by a tandem MBSII motif. To this end, a DNA fragment was PCR-amplified from VRE1::GUSint using the following primers:

MBSII-35Smin_fo CAGCTAAAGTTAGTTACGATGGCAAGACCCTTCCTC and GUS_re CCACACTTTGCCGTAATGAGTG (see Figure 5). After T/A cloning into pGemTeasy and sequencing, a 84-bp EcoRI-NcoI fragment comprising the MBSII-35Smin region was isolated and inserted into the corresponding sites in VRE1-35Smin::GUSint. Integrity of the plasmid (promoter sequence, GUS encoding region incl. 3′UTR, start codon, open reading frame were verified by sequencing).

A 449 bp Promoter region of Rd29A (At5g52310) was PCR-amplified from genomic *Arabidopsis* Col-0 DNA with primers Rd29A_fo GAGAAGGATGTGCCGTTTGT and Rd29A_re CTCTGTTTGATCCATGGTCCAAAGAT, inserted into pGemTeasy by T/A cloning and sequence-confirmed. The promoter was placed as EcoRI-NcoI fragment upstream of the GUS start codon.

### 4.5. β-Glucuronidase Activity Assay, Histological

GUS activity was assessed in *N. benthamiana* leaves 5 (and 8) days after infiltration. Leaves were infiltrated with substrate solution [0.2% Triton X-100, 50 mM NaHPO_4_ buffer (pH 7.2), 2 mM potassium ferrocyanide, 2 mM potassium ferricyanide, 1 mM X-Gluc (5-bromo-4-chloro-3-indolyl β-d-glucuronide cyclohexamine salt—from 100 mM stock in dimethyl formamide)]. Samples were incubated at 37 °C over night. Chlorophyll was removed by replacing the staining solution with a ethanol/acetic acid mix (3:1) and incubation at 70 °C. Destained leaves were subsequently inspected under a light microscope.

### 4.6. β-Glucuronidase Activity Assay in Protein Extracts

*Arabidopsis* mesophyll protoplasts were isolated and transformed with purified plasmids according to [65,66]. (MBSII-GUS or Rd29a-GUS reporter construct with the effectors YFP, MYB44 or MYB44-REP). An additional aliquot of protoplasts was transformed with 35S::YFP to document transformation efficiency. One day after transformation, β-glucuronidase activity in protein extracts was determined as described in [65] using MUG (4-methylumbelliferyl-β-d-glucuronide) as substrate. Six replicate transformations were performed for each promoter/reporter combination.

### 4.7. Transient Expression in *Nicotinana benthamiana*

Leaves of 5–6 week-old *N. benthamiana* plants were infiltrated with agrobacteria (strain GV3101, carrying pSoup and the pGreen construct(s) of interest) according to [67]. To confirm necrosis formation triggered by MYB44-REP expression, infiltrations were also performed with 2-, 3- and 4-week-old plants.

### 4.8. UV Microscopy

To document transient transformation efficiency in infiltrated *N. benthamiana*, YFP expression studies were conducted at a UV microscope (Leica DM5500B, Vienna, Austria), equipped with excitation/emission filters: BP450–450 nm/LP515 nm as described previously [40].

### 4.9. Protein Extraction and Immunoblot Analysis

*Arabidopsis thaliana* (Col-0) seedlings or *Nicotiana benthamiana* leaf discs were snap-frozen in liquid nitrogen. Proteins were extracted as previously described [40].

## 5. Final Conclusions: MYB44 Manipulation for Optimizing Plant Stress Tolerance

Ectopic expression of MYB44 has been shown to improve salt and drought stress tolerance in soy [41]. As our results suggest, this tolerance-improving effect is most likely attributable to transcriptional activation genes involved in the prevention of excessive ROS accumulation. Therefore, a promising strategy to further enhance the tolerance-improving properties of MYB44 involves mutation or deletion of its latent repressor domain and/or fusion to a transcriptional activation domain. Expressing such MYB44 variant under the control of stress-responsive promoters presents a means of minimizing undesired side effects (e.g., growth retardation) frequently encountered with constitutive overexpression strategies.

## Supplementary Information
