# Supplementary material for: Dominant Repression by Arabidopsis Transcription Factor MYB44 Causes Oxidative Damage and Hypersensitivity to Abiotic Stress
Source: Int J Mol Sci. 2014 Feb 13;15(2):2517–37. doi: 10.3390/ijms15022517 (PMC3958865; doi:10.3390/ijms15022517)
Supplement: Supplementary file 1 [file ijms-15-02517-s001.pdf]

Supplementary Information

Figure S1. Transient transformation in *N. benthamiana*—domumentation of transformation efficiency and successful expression of the MYB44 and MYB44-REP transgene. Leaves were infiltrated with *Agrobacteria* carrying the MBSII-GUS reporter and a construct for constitutive expression of YFP, MYB44-myc or MYB44-REP. Five days after infiltration, YFP expression was observed by UV microscopy. (**A**) MYB44 and MYB44-REP were detected in leaf protein extracts via immunoblot analysis using antibody directed against the myc epitope tag; and (**B**) A non-specific band is indicated by a dashed arrow. Two specific immunoreactive bands were observed.

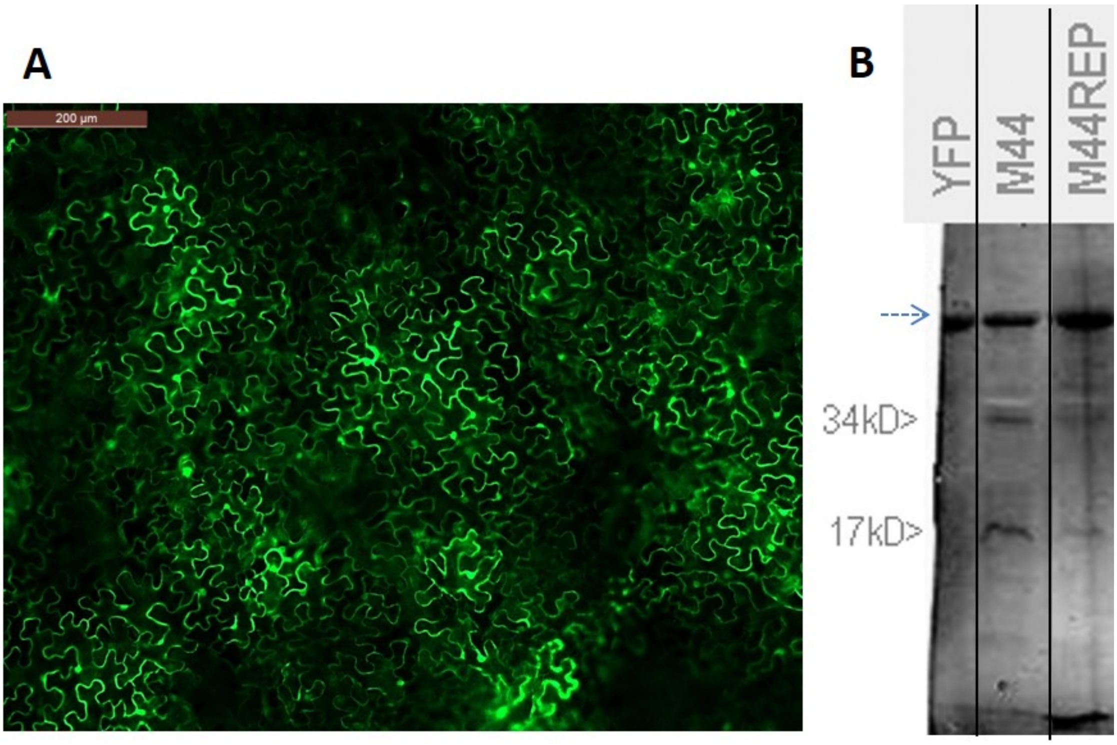

© 2014 by the authors; licensee MDPI, Basel, Switzerland. This article is an open access article distributed under the terms and conditions of the Creative Commons Attribution license (http://creativecommons.org/licenses/by/3.0/).
